# Diet, Stress and Mental Health

**DOI:** 10.3390/nu12082428

**Published:** 2020-08-13

**Authors:** J. Douglas Bremner, Kasra Moazzami, Matthew T. Wittbrodt, Jonathon A. Nye, Bruno B. Lima, Charles F. Gillespie, Mark H. Rapaport, Bradley D. Pearce, Amit J. Shah, Viola Vaccarino

**Affiliations:** 1 Departments of Psychiatry & Behavioral Sciences, Emory University School of Medicine, Atlanta, GA 30329, USA; mwittbr@emory.edu (M.T.W.); cgilles@emory.edu (C.F.G.); mark.h.rapaport@emory.edu (M.H.R.); 2 Department of Radiology, Emory University School of Medicine, Atlanta, GA 30332, USA; jnye@emory.edu; 3 Atlanta VA Medical Center, Decatur, GA 30033, USA; ajshah3@emory.edu; 4 Department of Medicine (Cardiology), Emory University School of Medicine, Atlanta, GA 30332, USA; kmoazza@emory.edu (K.M.); bruno.bezerra.lima@emory.edu (B.B.L.); lvaccar@emory.edu (V.V.); 5 Department of Epidemiology, Rollins School of Public Health, Emory University, Atlanta, GA 30322, USA; bpearce@emory.edu

**Keywords:** obesity, metabolic syndrome, serotonin, ghrelin, galanin, somatostatin, microbiome, brain, stress disorders, posttraumatic, child abuse, depressive disorder, diet, nutrition, cardiovascular disease, myocardial ischemia, coronary artery disease, Mediterranean diet

## Abstract

Introduction: There has long been an interest in the effects of diet on mental health, and the interaction of the two with stress; however, the nature of these relationships is not well understood. Although associations between diet, obesity and the related metabolic syndrome (MetS), stress, and mental disorders exist, causal pathways have not been established. Methods: We reviewed the literature on the relationship between diet, stress, obesity and psychiatric disorders related to stress. Results: Diet and obesity can affect mood through direct effects, or stress-related mental disorders could lead to changes in diet habits that affect weight. Alternatively, common factors such as stress or predisposition could lead to both obesity and stress-related mental disorders, such as depression and posttraumatic stress disorder (PTSD). Specific aspects of diet can lead to acute changes in mood as well as stimulate inflammation, which has led to efforts to assess polyunsaturated fats (PUFA) as a treatment for depression. Bidirectional relationships between these different factors are also likely. Finally, there has been increased attention recently on the relationship between the gut and the brain, with the realization that the gut microbiome has an influence on brain function and probably also mood and behavior, introducing another way diet can influence mental health and disorders. Brain areas and neurotransmitters and neuropeptides that are involved in both mood and appetite likely play a role in mediating this relationship. Conclusions: Understanding the relationship between diet, stress and mood and behavior could have important implications for the treatment of both stress-related mental disorders and obesity.

## 1. Introduction

The relationship between diet and behavior has long been a topic of interest. This includes the effects of diet on both mental and physical health, as well as related topics of the role of stress and obesity in these processes [1]. Dietary modification can prevent the development of cardiovascular disease (CVD) and diabetes [2], and stress-related mental disorders, including major depression and posttraumatic stress disorder (PTSD), are associated with an increased risk for CVD [3,4,5,6,7,8], although the mechanisms of these interactions are not well understood [9,10]. Specifically, there is a limited understanding of how diet affects mental health, and the way outcomes of unhealthy diet such as obesity interact with stress-related psychiatric disorders.

**Figure 1:**
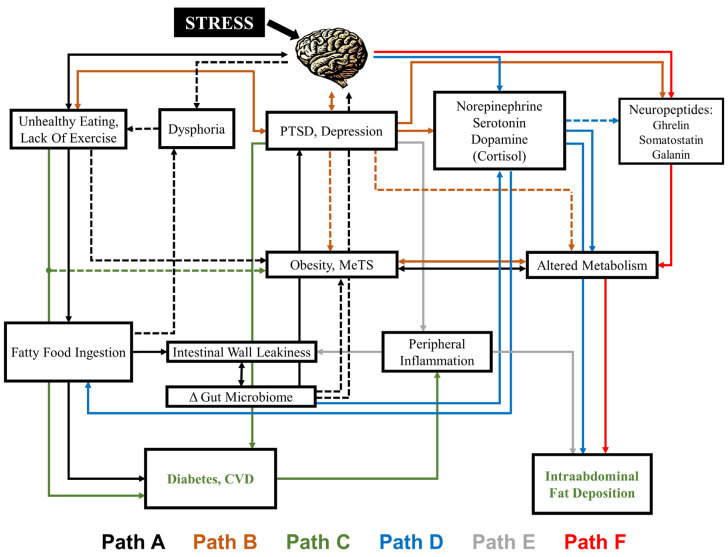
The complex relationship between diet, obesity and behavior. Stress acts through the brain to both affect eating and exercise behaviors (Path A) and stress-related psychiatric disorders including posttraumatic stress disorder (PTSD) and depression (Path B), both of which can lead to changes in metabolism, metabolic syndrome (MetS) and obesity (Paths A and B). Binary relationships also exist between unhealthy eating and PTSD/depression and the brain (i.e., both in turn lead to changes in brain function). Unhealthy eating can result in diets high in saturated fat (fatty food ingestion) (Path A) that can affect mood (dysphoria) as well as leakiness of the intestinal wall (Path A), which can lead to changes in the gut microbiome which modulate obesity, MetS and metabolism (Path A), as well as feeding back on the brain (Path A) to influence mood (dysphoria). Physical disorders including cardiovascular disease (CVD) and diabetes (Path C) and physical factors such as intra-abdominal fat (Path C) are affected by stress and related to PTSD and depression. A complex system of neurotransmitters (norepinephrine, serotonin, dopamine) (Path D), inflammatory markers (Path E) and neuropeptides (ghrelin, somatostatin, galanin) (Path F) present in the gut and brain are also influenced by stress via the brain, influence the gut microbiota and physical disorders and factors in a binary fashion and in turn regulate both feeding behavior and psychiatric disorders. Within the figure, the line color indicates the path, with dashed lines indicating primary pathways and solid lines indicating secondary pathways.

The relationship between these factors is often bidirectional. For example, changes in diet may influence psychiatric disorders through direct effects on mood, while the development of psychiatric disorders can lead to changes in eating habits [11]. Figure 1 shows the myriad and complex relationships that can exist between diet and psychiatric symptoms. In Path A in the figure, stress can act through the brain to cause an increase in over-eating [12], including binge-eating [13], and a reduction in exercise that in turn leads to obesity and/or metabolic syndrome (MetS, defined as increased blood pressure, and blood sugar, excess waist body fat and elevated cholesterol or triglyceride levels), which may in turn lead to disorders such as depression due to functional and/or social impairments [14]. In Path B, stress-related psychiatric disorders develop (PTSD, depression) that are associated with changes in metabolism and obesity [15]. Path C acts through physical disorders such as cardiovascular disease (CVD) and diabetes, that may come by way of PTSD and depression, and are related to those disorders in a bidirectional relationship or perhaps through shared pathways. Stress-induced over-eating leads to obesity, which in turn can be associated with changes in neurotransmitters, neuropeptides, and inflammatory factors that are present in both the gut and the brain and have effects on both mood and subsequent eating behaviors [16,17]. Path D shows the effects of neurotransmitters (e.g., serotonin) and neurohormones (cortisol), inflammatory factors are shown in Path E, and neuropeptides (ghrelin, galanin) reviewed below are shown in Path F. Finally, returning to Path A, changes in diet can affect the gut microbiome, which can have effects on mood, and is involved in a complicated bidirectional interaction between brain and inflammatory function as well as the abovementioned neurotransmitters and neuropeptides [18].

This paper reviews possible mechanisms by which diet and obesity can affect mental health and the brain, and the complex interplay between these different factors. We review the effects of diet on mood, with feelings of dysphoria or symptoms of depression most relevant within this context. Factors such as ingestion of fatty foods, as reviewed below, may impact feelings of dysphoria, which is relevant to the topic. Many studies reviewed below examined depressive symptoms in populations that are not suffering from psychiatric disorders, such as pregnant women, or looked at populations such as the elderly in the community at risk for nutritional deficiencies. These studies of depressive symptoms are to be differentiated from studies of populations that meet the criteria for major depression, which together with PTSD are more relevant to the practice of clinical psychiatry. Another relevant topic to effects of diet on mental health is the area of supplements and specific food products, including polyunsaturated fats (PUFAs), which have been the subject of a variety of clinical trials as both treatments and preventative agents for depression.

## 2. Materials and Methods

The literature was reviewed from 1980 through June of 2019 using PubMed and Psych info with keywords diet; obesity; stress disorders; posttraumatic; stress; depressive disorders; depression; inflammation; and polyunsaturated fatty acids. Relevant articles were reviewed and included here. We included both cross-sectional studies and cohorts (retrospective and prospective) that investigated the effects of diet on mood, including depression and dysphoria. This review focused on stress-related psychiatric disorders including major depression and PTSD, as defined in the Diagnostic and Statistical Manual (DSM-5) [19]. These are not the only two psychiatric disorders influenced by stress, of course, but they have been the focus of the bulk of the research on diet and stress, and related topics such as obesity and metabolism. Mental disorders are considered synonymous with psychiatric disorders, and, for the purposes of this review, refer to PTSD and depression, which are also referred to as stress-related psychiatric conditions or mental disorders, and mental health is considered as the absence of these conditions, again for the purposes of this review. Mood entails changes in emotions including depressed or sad emotions, or depressive symptoms, which may or may not be related to the DSM-5 diagnosis of major depression, but is referred to here as relevant to understanding the effects of stress and diet on behavior.

### 2.1. Effects of Diet on Health

In the past forty years, there has been a remarkable increase in worldwide obesity [20,21], which has been associated with an increase in type-2 diabetes and cardiovascular disease [22,23]. The increase in obesity is likely secondary to an expansion of the Western diet, which includes higher amounts of omega-6 relative to omega-3 fatty acids relative to other diets, largely through the substitution of carbohydrates in the form of grains for leafy plants, represented by ubiquitous processed foods [24]. Factors including an increase in perceived stress and a shift in the labor force from manual labor to sedentary occupations have also likely contributed to overeating (including binge eating) and the growth in obesity [2,25]. After a long period of no change, the prevalence of obesity in the United States doubled every decade in the 1980s and 1990s, and now one third of Americans are afflicted by obesity and another one third overweight [26]. Obesity is associated with a 44% increase in risk for myocardial infarction, with an even more important association with abdominal fat that further contributes to systemic inflammation [27,28]. From 2000 to 2010, the prevalence of type-2 diabetes increased by 46%, from 151 million to 221 million world-wide [29], with more recent estimates showing 442 million persons to be affected by type 1 and type 2 diabetes worldwide [30], an effect largely driven by obesity [31,32,33,34]. Ethnic Chinese who emigrated from their homes in China, where the prevalence of diabetes was 2%, to countries where Western diets were more prevalent, such as Mauritius, experienced an increase in diabetes prevalence to 15% [29]. Modification of diet can reduce risk of diabetes [35] and cardiovascular disease [36].

One dietary approach that has been particularly well researched is the Mediterranean diet. This diet is rich in nuts, vegetables and fruit, and low in meat, with moderate consumption of red wine, and substitutes of unsaturated fats (olive oil) for saturated and monounsaturated fats (butter, animal fat). Originally described in Italians living in the countryside, the diet was noted to be associated with long life spans and low rates of cardiovascular disease. Studies have confirmed that the Mediterranean diet is associated with sustained weight loss [37], with a beneficial effect on cardiac autonomic function, reductions in peripheral inflammation, and reduced rates of cardiovascular disease, stroke, cancer, and mortality [1,37,38,39,40,41]. The beneficial effects of diet on health have led to speculation that diet modification may be beneficial for symptoms of depression.

### 2.2. The Influence of Diet on Mood and Psychiatric Disorders

The relationship between diet and mental disorders is complex. Studies have shown an increase in depression in obese individuals [14,42,43] that is greater in the presence of MetS [14]. As outlined above, stress can lead to an increase in overeating [16,44,45,46], obesity [12,47,48,49,50] and psychiatric disorders, including PTSD and depression [15]. Depression in obese individuals may be due to psychological issues related to self-consciousness about appearance, common factors such as a history of childhood abuse [12,47,48,49,50], or factors such as diet-induced changes in the gastrointestinal microbiome with effects on the brain [51,52]. Early trauma associated with obesity in adulthood is likely due to a resetting of metabolism, independent of changes in eating habits related to dysphoria and other emotional disturbances [12,53]. Binary relationships also exist between these variables as well; for instance, stress causes changes in eating habits, leading to obesity and MetS, which in turn can exacerbate depression.

Diet can also have direct effects on mood. The effect of diet on brain function is one pathway through which diet can affect mood and the development of psychiatric disorders [42,43,44,45]. Fats are known to interfere with the synthesis of serotonin, a key brain neurotransmitter implicated in the development of depression, while proteins have the opposite effect [42]. Therefore, a high fat diet has been hypothesized to cause mood disorders (while a low-fat diet would have the opposite effect) [54]. Research studies have in fact shown that high fat foods can lead to transient changes in mood [55], possibly via signaling of gut flora that is perceived by the brain [45]. Intake of fats leads to feelings of sleepiness that are not related to the food alone [56,57]. Uncontrolled observational studies have shown a reduction in fats in the diet in depressed patients who have a resolution of depression [58] and a relationship between symptoms of anxiety and depression, and a low carbohydrate, high fat diet [59]. Survey studies showed that patients consuming a diet similar to the Mediterranean diet had a reduced risk for the development of depression [60]. In summary, the relationship between diet, stress and psychiatric disorders is complex and likely bidirectional, with diet affecting psychiatric symptoms and psychiatric symptoms affecting diet with interactions with stress and obesity.

Studies in populations without the diagnosis of depression on the effects of dietary interventions have been mixed [11,61]. Although some studies showed a reduction in depressive symptoms in healthy insurance company employees with a vegan diet [62], elderly persons at risk for malnutrition [63,64], and women with breast cancer [65] with nutritional interventions and/or diets, other studies in healthy subjects showed no effects of a low carbohydrate high fat compared to a high carbohydrate low fat diet [66] or a low-fat diet versus no intervention [67]. Studies of dietary interventions with and without exercise training aimed at weight loss for obese and overweight individuals have in general shown improvements in well-being and quality of life without clinically significant reductions in symptoms of depression [68,69,70,71]. Similarly, studies of the Mediterranean diet did not show a reduction in symptoms of depression in populations without the clinical diagnoses of depression, although there were improvements in factors such as quality of life or self-reported vigor in some studies [72,73,74,75,76,77].

Studies have looked at the effects of dietary interventions in patients with depression. Two studies included veterans identified as having some symptoms of depression and exposure to psychological trauma who were randomized to a diet education group or problem-solving therapy (PST). In one sample, treatment with PST was associated with greater improvement in mental health as measured with the SF-36 with no difference between groups in depressive symptoms measured with the Beck depression inventory (BDI) [78]. A second study with a similar sample failed to find an improvement in depression with diet training [79]. The Supporting the Modification of lifestyle in Lowered Emotional States (SMILES) trial was performed in patients with clinical depression. In this study, a modified Mediterranean Diet intervention was associated with improvement in depression as measured with the Montgomery Asberg Depression Rating Scale (MADRAS) compared with a social support intervention which matched the number and length of visits [80].

In addition to depression, recent studies suggested a relationship between sleep duration and Mediterranean Diet. In the MESA Sleep ancillary study, a higher adherence to a Mediterranean-type diet was associated with a 43% greater likelihood of achieving 6–7 h of sleep per night compared to 6 h [81]. Data from the Seniors-ENRICA and Hellenic Longitudinal studies also found a lower likelihood of poor sleep quality for those who were following a Mediterranean diet [82,83]. Finally, a study performed in France showed that adherence to a Mediterranean diet predicted reduced risk of insomnia [84].

In summary, there is not strong evidence for an effect of dietary intervention on symptoms of depression, although effects are possible in patients with the clinical diagnosis of depression.

### 2.3. Diet Interventions for Depression

The use of dietary supplements for the treatment of depression has been the subject of intensive investigation [11] (Table 1). Omega-3 polyunsaturated fatty acids (n-3 PUFAs) are highly concentrated in fish oil, especially from mackerel, salmon, herring and sardines. (n-3 PUFAs)contain more double carbon bonds than saturated fats, such as butter and animal fats, and are more likely to be solid than liquid at room temperature. n-3 PUFAs, including eicosapentaenoic acid (EPA) and docosahexaenoic acid (DHA), have beneficial effects on health, including reduction in cardiovascular disease risk, improved cognition, enhancement of neuroplasticity, and protection against neuronal injury [85,86,87,88,89]. n-3 PUFAs are included in the category of eicosanoids, which bind to intra-nuclear receptors and regulate gene transcription, making them part of the nuclear receptor superfamily, which includes steroids, thyroid hormone, and retinoids, all of which have been linked to neuropsychiatric syndromes [90]. These studies suggested that n-3 PUFAs may have benefit in the treatment and/or prevention of depression.

Patients with depression have an increase in inflammation [91,92,93] and n-3 PUFAs have an inhibitory effect on this system, which suggests another potential beneficial effect for this disorder [94]. Inflammasomes are multi-protein complexes that detect signals in the inflammation pathway and activate pro-inflammatory cytokines [95]. Danger-associated molecular patterns (DAMPs) induced by stress are recognized by pattern-recognition receptors (PRRs), which trigger production of interferon alpha and beta and pro-inflammatory cytokines. Families of PRRs that are components of the inflammasome complex include nucleotide-binding domain, leucine-rich repeat containing proteins (NLRs, or NOD-like receptors). These induce caspase-1 and the inflammatory response and play a role in diseases involving inflammation [96], including PTSD and depression [92,97]. Specialized proresolving mediators (SPMs), including resolvins, lipoxins, maresins, and protectins, act as brakes on the inflammatory response and are released at the time of the initial inflammatory response [98,99,100]. Resolvins D1 and D2, derived from DHA, act through the mammalian target of rapamycin complex 1 (mTORC1) in the medial prefrontal cortex and hippocampus to mediate anti-inflammatory and antidepressant effects [101,102]. Similar effects are seen with resolvins E1 and E2, which are derived from EPA [103,104]. Studies showed an increase in the inflammasome NLRP3 and caspase-1 in blood cells of patients with major depression, with associated increases in interleukin 1β (IL-1β) and IL-18, which correlated with depression severity [105]. NLRP12 was also found to be increased in Vietnam veterans with depression and coronary artery disease (CAD) [106]. Other studies showed an increase in myeloperoxidase [107].

Patients with depression also show alterations in endogenous n-3 PUFAs that may be linked to inflammatory alterations in these patients [11,54]. Patients with depression had higher blood concentrations of omega-6 relative to omega-3 fatty acids compared to individuals without depression [108], and depressed patients with low DHA and an elevated omega-6/omega-3 ratio in blood had an increased risk for suicide [109]. Elevated baseline inflammatory cytokines in patients with major depression predicted both risk for MetS [110] as well as a positive response to PUFA treatment for depression [94]. These studies suggested a complex but potentially relevant relationship between depression, inflammation, and n-3 PUFAs.

Studies have looked at n-3 PUFAs delivered as fish oil or DHA and EPA in comparison to placebo in several at risk non-depressed populations (Table 1). Hemodialysis patients randomized to a combination of DHA and EPA or placebo showed a significant reduction in depressive symptoms as measured with the BDI after four months [111]. EPA, but not DHA, resulted in a significant reduction in the number of patients who developed depression following treatment with interferon alpha for hepatitis C compared to placebo, although both EPA and DHA resulted in a significant delay in the onset of depressive symptoms [112]. DHA and/or EPA has not been found to result in significant decreases in symptoms of depression in patients with a history of ischemic stroke [113], mild cognitive impairment (MCI) [114], hyperlipidemia [115], or CAD [116,117]. Similarly, administration of DHA and/or EPA to pregnant and/or postpartum women did not result in a decrease in symptoms of depression [118,119,120,121]

Clinical trials of n-3 PUFAs have been conducted in patients with the diagnosis of depression. Randomized treatment with n-3 PUFAs in the form of fish oil, EPA and/or DHA compared to placebo showed a reduction in symptoms in patients with depression [11,54,92,107,122,123,124,125,126,127,128,129,130,131,132,133,134,135,136,137,138]. Patients with major depression and elevations in inflammatory biomarkers showed a stronger response to EPA (but not DHA) treatment compared to depressed patients without elevations in inflammatory biomarkers [94]. Other studies, however, did not show improvement in patients with depression [127,136,139,140,141,142,143,144,145]. Two studies of DHA and/or EPA added to the antidepressant sertraline in patients with depression and CAD or at risk for CAD showed no improvement compared to sertraline plus placebo [146,147]. Overall, studies are suggestive if not definitive, with a pattern of greater effect for EPA, especially when used as an adjunct to antidepressants, and in patients with more severe depression and/or biomarkers such as elevated inflammation.

Homocysteine is another dietary component that has been studied in relation to depression. Homocysteine is a sulfur-containing amino acid that is involved in carbon transfer reactions and is part of the B-12 and folate metabolic pathway [148]. Homocysteine can receive a methyl group from 5′-methyltetrahydrofolate and become re-methylated to methionine, the immediate precursor of S-adenosylmethionine (SAMe), a donor of methylation reactions involved in the synthesis of DNA, proteins, phospholipids, neurotransmitters and polyamines relevant to depression, including dopamine, norepinephrine and serotonin in the brain. Some studies have shown efficacy for SAMe in the treatment of depression [149].

Elevated homocysteine concentrations can result from folate or B12 deficiency. Several studies have found a relationship between elevated homocysteine levels and/or low folate and B vitamins and depression [150,151,152]. Diets deficient in folate and vitamins B6 and B12 are also associated with depression [153]. Since the Mediterranean diet is rich in folate and B12, it could possibly reduce the risk of depression through correction of this nutritional deficiency. Consistent with this, adherence to the Mediterranean diet was associated with a reduction in circulating levels of homocysteine [154].

Studies in patients with depression supported a link between elevated homocysteine and low folate and Vitamin B12 and depression. Low baseline folate and vitamin B12 [155] and elevated homocysteine [155,156] predicted subsequent development of depression, and elevated homocysteine correlated with a past history of depression in men (but not women), even after adjusting for health-related behaviors [157]. Low folate (but not low vitamin B12 or elevated homocysteine) has been shown to be a predictor of poor response to antidepressants [158,159,160,161], as well as risk of relapse, in patients with depression [159]. Treatment with folate and/or vitamin B12 in patients with elevated homocysteine compared to placebo in healthy older adults resulted in significant decreases in homocysteine [162,163], but no improvement in cognition [162] or symptoms of depression [163,164] (Table 1). Long-term supplementation with folate and vitamin B12 did not prevent the development of depression in healthy women [165]. Studies in patients with depression showed that folate and/or Vitamin B12, including as an augmentation to antidepressants, resulted in improved symptoms of depression in some studies [166,167,168,169,170] but not others [164,171,172]. Greater reductions in depression were seen in depressed patients with the MHTFR polymorphism [167,173]. Supplementation of antidepressants with folate and/or vitamin B12 in one study reduced the risk of relapse after successful remission [172]. Not all studies, however, have found an association between homocysteine and depression [174,175,176,177,178,179]. The population-based Rotterdam Study found an association between depression and homocysteine; however, the association was reduced after controlling for cardiovascular disease and functional disability [152]. These inconsistent results may be due to factors such as variations in diet and vitamin supplementation, which can affect folate intake, which influences homocysteine concentrations.

The Dietary Inflammatory Index has been developed in recent years to characterize an individual’s diet on a continuum from anti- to pro-inflammatory. Numerous studies investigating the role of Dietary Inflammatory Index on depression [180,181,182,183]. Results from the Whitehall II Study show a positive association between this index and recurrent depressive symptoms in women but not men [181]. Similar findings were observed in the Australian Longitudinal Study on Women’s Health, where women with the highest inflammatory diet had an approximately 20% higher risk of developing depression compared to those with the lowest pro-inflammatory diet [180]. In another cohort study of 15,093 university graduates, participants in the highest quintile of Dietary Inflammatory Index were found to have a 47% increased risk of developing future depression compared to those in the lowest quintile [182].

Studies on the relationship between diet and depression have limitations. Many of the studies reviewed above and in Table 1 involve populations without clinical depression and have considerable variability from study to study. Although it can be argued that some of these groups should be the subject of study because of potential risk for the development of depression, such as pregnant women, or patients with stroke, cancer, renal failure, CAD or MCI, they do not have equivalent relevance to the practice of clinical psychiatry as patients with the current diagnosis of major depression. Overall, findings from these studies suggest that although diet and/or supplements such as PUFAs may enhance “vigor” or quality of life, there is limited evidence for the prevention of or reduction in the development of symptomatic depression. Studies that did report positive results in non-clinical samples also had important limitations. For instance, the study of employees of an insurance company (the GEICO study) randomized to a vegan diet versus control used a design where group assignment occurred at company sites rather than on an individual level, so one company site was having regular meetings with dieticians, which were presumably discussed amongst employees, while the other site received nothing. Additionally, the study involved healthy individuals, used a measure not validated as a measure of clinical depression, and was performed by an advocacy group whose mission includes promotion of a plant-based diet [62]. Similarly, in a study of women with breast cancer, a dietary intervention induced a reduction in depression that was related to a one-point mean change in a 30-point scale (CES-D), with no change in mental health function, which although statistically significant, was of questionable clinical significance [65]. Obese patients with knee pain assigned to diet or exercise or no intervention showed a statistically significant improvement in depression measured with the Hospital Anxiety and Depression Scale (HADS). The improvement, however, was less than one point on a 21-point scale, which again was of questionable clinical significance [70]. Another study in obese and overweight post-menopausal women randomized to weight loss diet and/or exercise versus no intervention showed an improvement in depression as measured with the Brief Symptom Inventory (BSI) in the exercise and diet group, but not in the diet alone group [184]. These effects did not reach significance after correction for multiple comparisons, however, and involved a 1.7-point change in the BSI (4% change from baseline) of questionable clinical significance. EPA, but not DHA, resulted in a significant reduction in the number of patients who developed the diagnosis of depression as measured with the Mini following treatment with interferon alpha for hepatitis C compared to placebo, although both EPA and DHA resulted in a significant delay in the onset of depressive symptoms. Neither resulted in a significant reduction in depression compared to placebo on the Hamilton Depression Rating Scale (HDRS) [112]. Therefore, it can be seen that in non-clinical studies, most failed to show an effect of dietary intervention on depression, and if they did it was of questionable clinical significance. Studies in patients with the clinical diagnoses of depression also suffered from limitations. The Supporting the Modification of lifestyle in Lowered Emotional States (SMILES) trial was performed in patients with clinical depression. A modified Mediterranean diet intervention was associated with improvement in depression, as measured with the MADRAS compared to a social support intervention which matched the number and length of visits [80]. This study was criticized, however, for using recruitment materials implying a belief in the positive effects of diet on depression, including ads that had pictures of men made out of fruits [185]. This criticism is similar to that of the GEICO study, in that a bias for dietary change over medications may have been a confounding factor. In fact, nationalistic or cultural biases need to be considered as well. For example, we organized a conference on Mediterranean diet in conjunction with a Southern European country, who presented us with a giant round of Pecorino Romano cheese courtesy of their government’s office for the promotion of national products. Similarly, findings supporting beneficial effects of an Indian diet fell apart after publication in *The Lancet*, when a site visit to the study location revealed only a few broken electrocardiogram machines in an abandoned building in the jungle and the claim that the original data from the study had been eaten by termites [186]. Even in the absence of financial or cultural bias, opinions run strong on the subject of diet and depression. For instance, a meta-analysis found no significant effect of PUFAs on depression after adjusting for possible publication bias, and concluded that positive effects were limited to small studies [187]. This paper was met with another meta-analysis that strongly disputed these conclusions [131]. There have in fact been a large number of meta-analyses, mostly concluding a positive effect for PUFAs on depression [131,132,133,134].

Although specific foods may have short-term effects on mood, overall the findings are equivocal for clinically significant effects of diet on mental health. Evidence for a link between obesity and depression is also inconsistent. Replicated findings in fact show a stronger link for MetS than obesity with depression [14,34]. It is questionable whether research on the effects of diet on mental health can be adequately controlled given the high expectancy bias for the effects of diet on mood, the inability to mask the nutrition interventions, and the psychosocial impact of multiple meetings in groups devoted to education about health and diet [185]. Effects of n-3 PUFAs on patients with the clinical diagnosis of depression are stronger. In fact, the American Psychiatric Association now recommends intake of 500 mg of omega-3 fatty acids per day in the diet for patients with depression.

### 2.4. Neurotransmitters and Neuropeptides Affected by Diet, Obesity and Psychiatric Disorders Related to Stress

Alterations in neurotransmitters and neuropeptides that are involved in stress, psychiatric disorders and/or appetite represent a possible mechanism by which stress may increase the risk of obesity and form a link between diet and stress-related psychiatric disorders. Tissues in the brain and the gut are both derived from ectodermal cells in fetal development, and share signaling pathways in common that can provide a link between stress, diet, obesity, and psychiatric disorders, including neurotransmitters (Pathway D in Figure 1) and neuropeptides (Pathway F in Figure 1). Serotonin is a neurotransmitter present in the brain and the gut that regulates a wide variety of relevant behaviors and physiological functions, including the regulation of anxiety, arousal, vigilance, aggression, mood, impulsivity, sleep, and food intake, as well as physical functions including cardiovascular, respiratory, motor output, neuroendocrine secretion, and analgesia [188,189]. Stress is associated with alterations in serotonin function in the medial prefrontal cortex and other brain areas involved in the stress response [190,191] and altered serotonin function is associated with both depression and PTSD [192,193]. Cortisol plays a critical role in stress, is altered in PTSD, and is associated with increased deposition of intraabdominal fat [194]. Norepinephrine release in the brain is an important part of the behavioral response to stress [195,196]. The majority of norepinephrine cell bodies are located in the brain stem, in the locus coeruleus region of the pons, with axons that extend throughout the rest of the brain, and are activated by stress, leading to fear and anxiety behaviors [197,198,199,200,201,202,203,204]. Alterations in noradrenergic and peripheral sympathetic function play a role in the maintenance of symptoms of both depression and PTSD [193,195,196,205,206]. Norepinephrine also plays an important role in feeding behavior [207]. Dopamine is a neurotransmitter that is involved in a number of functions including the control of locomotion, cognition, affect, neuroendocrine secretion, reward systems including feeding behavior, and the stress response [208,209,210]. Dopaminergic innervation of the nucleus accumbens, the mesolimbic pathway, regulates feelings of pleasure, and deficits in this system could underlie feelings of a lack of pleasure or anhedonia in patients with stress-related psychiatric disorders [210]. Mesolimbic dopamine also underlies the rewarding properties of food and drives food seeking behaviors, although additional brain regions are also involved in this process [208].

Neuropeptides are another potential link between stress, diet and psychiatric disorders. Somatostatin is the major inhibitor of growth hormone (GH) secretion in the brain and is located in the paraventricular nucleus (PVN) of the hypothalamus, the amygdala, hippocampus, cerebral cortex, median preoptic area, nucleus accumbens, and other areas of the brain [211]. Somatostatin inhibits extinction, modulates sleep, food intake, locomotor activity, and memory function [212] and increases in response to stress [213]. Increased somatostatin levels in the CSF were seen in patients with PTSD [214]. Galanin is a peptide concentrated in brain areas involved in the stress response that mediates a number of physiological and behavioral functions, including learning and cognition, pain control, food intake, neuroendocrine control, and cardiovascular regulation, as well as depression and anxiety [215,216]. Alterations in galanin have been hypothesized to underlie depression [215,216]. Ghrelin is a peptide synthesized in the stomach and pancreas that is involved in the regulation of appetite and food digestion. Ghrelin levels rise before meals and stimulate food intake, and alterations in ghrelin function have been linked to obesity [217]. Stress increases ghrelin levels in the plasma [218,219], and ghrelin mediates the stress-induced increase in food intake associated with exposure to chronic stress [220] as well as behavioral responses to stress [219]. Ghrelin crosses the blood–brain barrier, where it acts through ghrelin receptors to stimulate the release of growth hormone in the basolateral nuclear of the hypothalamus and the amygdala, where it enhances fear learning with chronic stress [221]. In summary, a number of neurotransmitters and neuropeptides that are located in both the brain and the gut and that mediate both stress and feeding behaviors may be the link between stress, diet and stress-related mental disorders.

### 2.5. The Gut-Brain Connection

Recently, the role of the gastrointestinal microbiome in affecting brain function has been recognized, with possible implications for the development of mental disorders in the context of diet and metabolism [18,52]. The gastrointestinal flora is composed of a range of bacterial species that have beneficial effects for our digestion and other functions; these are also affected by factors such as stress and a high fat diet [222,223,224] and influence brain function through the release of various signaling molecules. Additionally, they interact with neurotransmitters and neuropeptides reviewed in the prior section, which can lead to changes in mood and stress reactivity [45,224,225,226,227]. High fat diets can lead to leakiness of the gut epithelium, resulting in the release of inflammatory factors and penetration of gut flora in the intestinal wall, which can further increase the risk of depression via alterations in signaling pathways leading to the brain [51,227,228]. Studies have shown that the gut biota can influence both risk for MetS, and via gut biota that synthesize a metabolite of dopamine risk for depression [229,230]. Studies have looked at the effects of the replacement of the bacterial flora with probiotics or other agents for the treatment of mood disorders, with some success [229,231]. In conclusion, the gut microbiome interacts with diet and the environment to affect mental disorders in complex ways that are imperfectly understood but represent a promising area for future research and possible interventions.

## 3. Conclusions

The relationship between diet, obesity, stress, and stress-related psychiatric disorders is complex. Overall, there appears to be a link between diets low in saturated fats and high in omega-3 polyunsaturated fats and reduced risk of both obesity, MetS and stress-related psychiatric disorders, as well as beneficial effects for other health outcomes. This favors a diet that is rich in fruits, nuts, and vegetables, and fish, as is seen in the Mediterranean diet. Specifically, fish oils are a rich source of omega-3 fatty acids, and likely have a beneficial effect on mental and physical health. There are a number of potentially confounding factors, however, such as increased healthy behaviors in those who adhere to specific diets that could contribute to a spurious association. Most studies have focused on dietary interventions such as fish oils or PUFAs in the treatment of depression or the prevention of symptoms of depression in at risk groups. There is not good evidence for dietary interventions for at risk groups, and given the enthusiasm for diet as an intervention, the possibility of confirmation bias cannot be ruled out.

Although a Mediterranean diet, in combination with other behavioral changes, was found to have beneficial effects on perceived stress and well-being, it has not been shown to specifically benefit depression. Some elements of the Mediterranean diet, such as omega-3 fatty acids (found in fish), have been found to be beneficial in some clinical trials. Meanwhile, the addition of folate to the diet (with reduction in homocysteine) is associated with decreased symptoms of depression. In countries such as the United States, folate is added to flour, so depression-related folate deficiency is no longer an issue clinically, although not all countries incorporate the addition of folate to food. Thus, clinicians are likely to continue to advocate for adherence to the Mediterranean diet or diets low in fat and high in n-3 PUFAs as part of a general program of health-promoting behaviors, including exercise. It cannot be assumed, however, that supplements like EPA and DHA will confer the same advantages as consumption of fish, whose dietary compilation they are designed to emulate.

The relationship between high saturated fat diets and mental disorders is more complex. It appears that intake of fat may have an acute effect on mood, leading to symptoms of anxiety and depression. In addition, resolution of depression was associated with a reduction in fat intake in some studies, although it is not clear if the reduction in fat led to resolution of depression, or whether resolution of depression led to improved eating habits. Finally, a healthy diet with reduction in obesity will likely have beneficial effects on mental health through improved feelings of wellness and self-esteem, in addition to the known association between obesity and depression. Observational studies of the effects of diet for weight loss and dietary interventions on depression and anxiety have had mixed results, and it is not clear if the science supports clear recommendations for dietary interventions, apart from the “does no harm” approach. Studies with the greatest effect were those where diet was paired with exercise training, which is significant since studies showed benefits of aerobic exercise equivalent to antidepressants for the treatment of clinical depression [232]. The strongest evidence for a role of diet intervention for depression was in the area of PUFA supplements, specifically higher doses of EPA, for patients with the clinical diagnosis of depression. Since there are essentially no side effects, its use as an adjunct to antidepressants should certainly be considered as part of a treatment armamentarium, although not a substitute for current treatments.

Future studies need to assess the relationship between diet and mental disorders. Future studies should emphasize a multi-disciplinary and integrated approach, the use of epidemiological research methods involving studies in the general population of lifestyles beneficial for health, application of rigorous clinical trial methodology, and promotion of healthy behaviors. Studies of interventions in large communities, including schools, are needed, as well the study of the relationship between diet and health in an international context. Finally, further research in the area of the gut biota, neurotransmitters and neuropeptides, and biological systems in the brain and gut will likely contribute to greater knowledge that can advance treatment for stress-related psychiatric disorders, as well as obesity and MetS.

## Figures and Tables

**Table 1:** Controlled Trials on Dietary Interventions for Depression.

Author-Year	Study	Population	Intervention	Sample	Outcome measure	Result	
Agarwal 2015	GEICO	Healthy employees	Vegan diet v no intervention	Randomized (*n* = 292)	SF-36	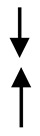	depression, anxiety, QOL
*Almeida 2014*	*B-VITAGE Adjunctive to antidepressants*	*Major depression >age 50*	*VB6/VB12/Fol v Pla*	*Randomized (n = 153)*	*MADRS*	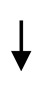	*relapse, MADRS NS*
Andreevna 2012	SU.FOLOM3	CAD patients	EPA/DHA v placebo	Randomized (*n* = 2501)	GDS	NS depression	
Assaf 2016	WHI Study	Women	Low fat diet v no intervention	Randomized (48,835)	CES-D	NS depression	
*Bedson 2014*	*FolATED Adunctive to antidepressants*	*Patients with depression*	*Fol v pla*	*Randomized (n = 475)*	*BDI, MADRS*	*NS depression*	
*Bot 2010*		*Patients with MDD & Diabetes*	*EPA v placebo*	*Randomized (n = 25)*	*MADRAS*	*NS depression*	
*Carney 2009*	*Adjunctive to antidepressants*	*Patients with CAD & depression*	*DHA/EPA v placebo*	*Randomized (n = 122)*	*HDRS, BDI*	*NS depression*	
*Carney 2019*	*Adjunctive to antidepressants*	*Patients with CAD or hi risk & depression*	*EPA v placebo*	*Randomized (n = 144)*	*HDRS, BDI*	*NS depression*	
*Coppen 2000*	*Adjunctive to antidepressants*	*Major depression*	*Fol v pla*	*Randomized (n = 127)*	*HDRS*	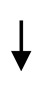	**depression**
*Da Silva 2008*	*Adjunct ±antidepressants*	*Patients with PD & depression*	*Fish oil v placebo*	*Randomized (n = 31)*	*MADRAS, BDI*	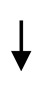	*MADRS depression, BDI NS*
De Koning 2016	B-PROOF	Community sample, older adults	Fol/VB12 v pla	Randomized (*n* = 2,919)	GDS	NS depression	
Doornbos 2009		Pregnant women	EPA/DHA v placebo	Randomized (*n* = 119)	EPDS	NS depression	
Endevelt 2011	Israel	Elderly at risk for malnutrition	5 visits with dietician & MD v booklet v nothing	Randomized (*n* = 127)	GDS	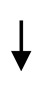	Depression with dietician & MD
Einvik 2010	Oslo Diet & Antismoking Study	Patients with hyperlipidemia	n-3 PUFAs v placebo v dietary counseling	Randomized (*n* =563)	HADS	NS depression	
Forster 2012	UK	Elderly in community	Diet intervention v supplement v placebo	Randomized (*n* = 217)	GDS	NS depression	
*Freeman 2008*	*Adjunctive to therapy*	*Perinatal MDD*	*DHA/EPA v placebo*	*Randomized (n = 51)*	*HDRS*	*NS depression*	
*Garcia Toro 2016*	*Adjunctive to antidepressants*	*Patients with depression*	*Med Diet adherenec*	*Observational (n = 273)*	*BDI*	*NS depression*	
Gharekhani 2014		Hemodialysis patients with depressive symptoms	DHA/EPA v placebo	Randomized (*n* = 54)	BDI	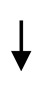	Depression
*Gertsik 2012*	*Adjunctive to antidepressants*	*Patients with depression*	*Citalopram + EPA/DHA/O3FA v Cit + pla*	*Randomized (n = 46)*	*HDRS*	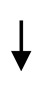	*depression*
*Grenyer 2007*	*±Adjunctive to antidepressants*	*Patients with depression*	*DHA/EPA (fish oil) v placebo*	*Randomized (n = 83)*	*HDRS, BDI*	*NS depression*	
Halyburton 2007		Overweight or Obese patients	Low carb high fat v high carb low fat diet	Randomized (*n* = 93)	POMS, BDI	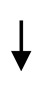	Weight in both groups, cognition low fat, NS depression
Hyypa 2003		Men with hyper- cholesterolemia	Simvistatin v Med Diet v placebo	Randomized (*n* = 120)	BSI	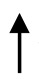	NS depression
Imayama 2011		Obese and overweight post-menopausal women	Weight loss diet, exercise v no intervention	Randomized (*n* = 439)	BSI	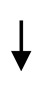	Depression diet/exercise, NS depression diet alone
*Jacka 2017*	*SMILES study*	*Patients with clinical depression*	*Med. Diet v Social Support*	*Randomized (n = 67)*	*MADRAS*	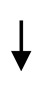	*Depression*
*Jazayeri 2008*		*Patients with clinical depression*	*Flu + EPA v flu + pla v EPA + pla*	*Randomized (n = 60)*	*HDRS*	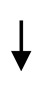	*Depression*
Jenkinson 2009		Obese patients with knee pain	Diet v exercise v no intervention	Randomized (*n* = 389)	HADS	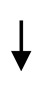	Depression
Kasckow 2014		Veterans with symptoms of depression	Diet Education Group v PST	Randomized (*n* = 45)	BDI, SF-36	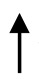	NS depression, GMH
Kasckow 2014		Veterans with symptoms of depression	Diet Education Group v PST	Randomized (*n* = 60)	BDI	NS depression	
Kwok 2019		Older MCI+ H-Hcy	VB12/fol v pla	Randomized (*n* = 279)	CDR, HDRS	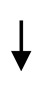	Depression
Llorente 2003		Obese and overweight post-menopausal women	DHA v placebo	Randomized (*n* = 439)	BSI	NS depression	
*Lesperance 2011*		*Patients with depression*	*EPA/DHA v placebo*	*Randomized (n = 432)*	*IDS*	*NS depression*	
Llorente 2003		Pregnant women	DHA v pla	Randomized (*n* = 99)	BDI		
Lucas 2009		Middle aged women with symptoms of depression	EPA/DHA v pla	Randomized (*n* = 120)	HDRS	NS depression	
Makrides 2010	DOMInO study	Pregnant women	DHA v pla	Randomized (*n* = 2399)	EPDS		NS depression
*Marangell 2003*		*Patients with depression*	*DHA v placebo*	*Randomized (n = 36)*	*MADRAS*		*NS depression*
*McMillan 2011*		*Young healthy women*	*Med Diet v no intervention*	*Randomized (n = 25)*	*POMS*	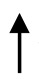	*NS depression, cognition* *. vigor*
*Mech 2016*		*MDD+ MTHFR*	*VB6/VB12 v pla*	*Randomized (n = 330)*	*MADRS*	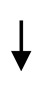	*depression*
*Mischoulon 2009*		*Patients with depression*	*EPA v placebo*	*Randomized (n = 35)*	*HDRS*	*NS depression*	
*Mischoulon 2015*		*Patients with depression*	*EPA v DHA v placebo*	*Randomized (n = 196)*	*HDRS*	*NS depression*	
*Mozaffari-Khosravi 2013*		*Patients with depression*	*EPA v DHA v placebo*	*Randomized (n = 81)*	*HDRS*	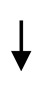	*Depression EPA, DHA NS depression*
Mozurkewich 2013	The Mothers, Omega-3 and Mental Health Study	Pregnant with symptoms of depression	EPA Fish oil v DHA fish oil v placebo	Randomized (*n* = 118)	BDI	NS depression	
*Nemets 2002*	*Adjunctive to antidepressants*	*Patients with MDD*	*EPA v placebo*	*Randomized (n = 20)*	*HDRS*	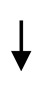	*Depression*
*Nemets 2006*		*Children with depression*	*EPA/DHA v placebo*	*Randomized (n = 28)*	*CDI, CDRS*	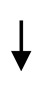	*Depression*
Nieman 2000		Obese women	Exercise and/or weight loss diet v wait list	Randomized (*n* = 91)	POMS		NS depression
Okereke 2015	WAFACS	Normal women	VB6/VB12/Fol v pla	Randomized (*n* = 4331)	Clin dx		NS depression
*Peet 2002*	*Adjunct to TAU*	*Patients with MDD*	*EPA v placebo*	*Randomized (n = 70)*	*HDRS, MADRAS*	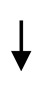	*Depression*
Poppitt 2009		Patients with Ischemic Stroke	Fish Oil with O3FA v pla	Randomized (*n* = 102)	GHQ	NS depression	
*Rees 2008*		*Perinatal MDD*	*DHA/EPA (fish oil) v placebo*	*Randomized (n = 26)*	*HDRS, MADRAS*	*NS depression*	
*Rondanelli 2010*		*Elderly women with depression*	*DHA/EPA v placebo*	*Randomized (n = 46)*	*GDS*	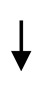	*depression*
Scheier 2005		Young Women with Breast Cancer	Nutrion group v Health Ed v TAU	Randomized (*n* = 252)	CES-D	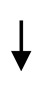	Depression with nutrition group
Serrano Ripoll 2015		Primary Care Patients	Diet & exercise instructions v control	Randomized (*n* = 273)	BDI	NS depression	
*Silvers 2005*		*Patients with MD in treatment*	*EPA/DHA (Fish oil) v placebo*	*Randomized (n = 77)*	*HDRS*	*NS depression*	
Sinn 2012		Patients with MCI	DHA v EPA v linoleic acid	Randomized (*n* = 50)	GDS	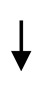	Depression
*Stoll 1999*		*Patients with Bipolar Disorder*	*EPA/DHA (Fish oil) v placebo*	*Randomized (n = 30)*	*Time to relapse (clinical)*	*Delayed relapse*	
*Su 2003*	*Adjunct to TAU*	*MDD*	*EPA/DHA v placebo*	*Randomized (n = 28)*	*HDRS*	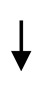	*depression*
*Su 2008*		*Pregnant women with MDD*	*EPA/DHA v placebo*	*Randomized (n = 36)*	*HDRS*	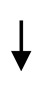	*depression*
Su 2014		Interferon patients	DHA or EPA v placebo	Randomized (*n* = 162)	Mini	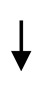	Depression onset
*Tajalizadekhoob 2011*	*Adjunct to TAU*	*Elderly with mild to mod depression*	*EPA/DHA (Fish oil) v placebo*	*Randomized (n = 66)*	*GDS*	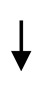	*depression*
*Tayama 2019*		*Patients with mild to mod depression*	*EPA/DHA v placebo*	*Randomized (n = 90)*	*BDI*	*NS depression*	
Toobert 2007	Med Lifestyle Program	Postmenopausal women with DM2	Med Diet & exercise v control	Randomized (*n* = 279)	CES-D	NS depression or QOL	
Wardle 2000		Patients with hyper- cholesterolemia	Low fat v Med Diet v wait list	Randomized (*n* = 176)	BDI, POMS	NS depression	

Controlled trials of the effects of diet and/or diet education on symptoms of depression. Studies in italics include trials of patients with clinical depression. BDI = Beck Depression Inventory; BSI = Brief Symptom Inventory; CAD=Coronary Artery Disease; CDI = Children’s Depression Inventory; Clin dx=clinician diagnosis of depression; CRD = Clinical Dementia Rating Scale; CDRS=Children’s Depression Rating Scale; CES-D = Center for Epidemiological Studies-Depression Scale; Cit = citalopram; DM2 = Type 2 Diabetes Mellitus; DOMInO=DHA to Optimize Mother Infant Outcome trial; EDPS = Edinburgh Postpartum Depression Scale; Flu = fluoxetine; Fol = Folate; GDS=Geriatric Depression Scale; GHQ = General Health Questionnaire; GMH = General Mental Health; HADS = Hospital Anxiety and Depression Scale; Hcy = homocysteine; H-Hcy = hyperhomocysteinemia; HDRS = Hamilton Depression Rating Scale; IDS = Inventory of Depressive Symptomatology; n-3 PUFAs = n-3 polyunsaturated fatty acids; MADRAS = Montgomery Asberg Depression Rating Scale; MCI = Mild Cognitive Impairment; MDD = Major Depression Disorder; Med Diet = Mediterranean Diet; Mini = Mini International Psychiatric Interview; MTHFR = methylenetetrahydrofolate reductase plymorphism; NS = non significant (no difference between study groups); O3FA = Omega-3 Fatty Acids; Pla = Placebo; POMS = Profile of Mood States; PST = Problem Solving Therapy; Rem = remission of depressive symptoms; SF-36 = Short Form 36 Items (Quality of Life (QOL), depression, anxiety); SMILES = Supporting the Modification of Lifestyles in Lowered Emotional States (trial); TAU = Treatment as Usual; VB12 = Vitamin B-12; 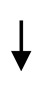 = decrease; 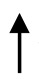 = increase; VB6 = Vitamin B-6WHI = Women’s Health Initiative.
